# Hepatitis B immunization strategies and its associated factors: A narrative review

**DOI:** 10.1002/hsr2.1748

**Published:** 2023-12-08

**Authors:** Emmanuel Ifeanyi Obeagu

**Affiliations:** ^1^ Department of Medical Laboratory Science Kampala International University Kampala Uganda

**Keywords:** adolescents, adults, blood, children, health workers, hepatitis B, immunization, liver cancer

## Abstract

**Background:**

Hepatitis B is a major public health concern as one third of the world's population is infected with hepatitis B virus (HBV). The risk of HBV infection remains primarily through percutaneous or mucosal contact with infected blood or body fluids.

**Aim:**

To update the society on hepatitis B immunization strategies and its associated factors.

**Materials and Methods:**

The review paper utilized different search engines such as Pubmed Central, Scopus, Web of Science, Google Scholar, and so on to conduct this review paper.

**Results:**

Improving immunity through general vaccination recommendations has reduced the number of chronic hepatitis B cases among children and adolescents, but an influx of infections from endemic countries has increased the number of chronically infected adults. This is due to immigration from endemic countries to Hepatitis less burden areas like developed countries. Introduction of infant immunization programs in some countries has reportedly reduced the prevalence of HBV infection and reduced the incidence of liver cancer in children and young adults.

**Conclusion:**

While hepatitis B vaccination coverage is low, a significant number of people are not adhering to full hepatitis B vaccination posing a potential hazard to health.

## 1 INTRODUCTION

Hepatitis B infection is an ancient Bronze Age disease suspected to be an infectious agent in the 1950s, then first described as an Australian antigen in the 1960s and then discovered by electron microscopy in the 1970s. Vaccination against hepatitis B was introduced in the 1980s.^1^ Hepatitis B is a major public health concern as one third of the world's population is infected with the hepatitis B virus (HBV). Based on the prevalence of hepatitis B surface antigen (HBsAg), different regions of the world are classified as having high (>8%), moderate (2%–7%), or low (<2%) HBV prevalence. The seroprevalence among healthcare workers is two to four times higher than that of the general population.^2^, ^3^, ^4^ The risk of HBV infection remains primarily through percutaneous or mucosal contact with infected blood or body fluids. If the HCW is an apprentice, intern, or just a student, the risk may be even greater due to lack of experience, inadequate training, or apparent negligence.^1^ Vaccination, safe handling of infectious materials, proper sterilization of medical equipment, and proper waste disposal can break the chain of transmission of hepatitis B infection. However, research shows that there is a clear knowledge gap among healthcare practitioner trainees regarding their occupational risk of HBV infection.^5^

## 2 GLOBAL BURDEN OF HEPATITIS B

HBV infection is a major global public health problem and requires high priority for prevention and control. More than 2 billion people are exposed to HBV worldwide, of which an estimated 387 million are currently chronically infected, and approximately 10 million new carriers emerge each year. About 17% of carriers die from HBV infection, with an overall annual mortality rate of about 1 million.^6^

It is estimated that between 700,000 and more than 2 million people in the United States have chronic hepatitis B infection.^7^ The number of chronically infected people worldwide and in the United States is difficult to estimate because the disease is often asymptomatic, leading to underdiagnosis, and passive surveillance often leads to underreporting. Improving immunity through general vaccination recommendations has reduced the number of chronic hepatitis B cases among children and adolescents, but an influx of infections from endemic countries has increased the number of chronically infected adults in the United States. This is due to migration from hepatitis B endemic countries to fewer burden countries. It is estimated that up to 70% of HBV infections in the United States occur in foreign‐born individuals. Each year, 40,000–45,000 people legally travel to the United States from HBV‐endemic countries where chronic HBV infection rates exceed 2%. An estimated 3.9 million foreign‐born people from East Asia and sub‐Saharan Africa currently live in the United States.^7^

The introduction of infant immunization programs in some countries has reportedly reduced the prevalence of HBV infection and reduced the incidence of liver cancer in children and young adults. Member States of the World Health Organization (WHO) are working towards the goal of eliminating HBV infection and its effects globally. A key strategy is infant immunization programs to achieve direct and collective protection. The WHO has set an eradication target of reducing the prevalence by 90% by 2030, but only a minority can reach this target.^8^

### 2.1 Hepatitis B vaccination

Current systems to combat HBV infection include comprehensive vaccination of all infants at birth, as well as high‐risk teens and adults, such as business sex professionals.^9^ Three doses of the HBV vaccine are given over 90% of adults and over 95% of them to young people. CDC mandates that vulnerable individuals who are screened for HBV disease, if properly identified, are able to receive significant HBV vaccination results at similar treatment visits.^10^

The U.S. Food and Drug Administration (FDA) approved the first antibody against hepatitis B infection (HBV) in 1982 for worldwide newborn vaccination programs.^9^ From that point on, protective and effective HBV antibodies became the best way to combat her HBV disease and its effects worldwide. Currently, up to 400 million people worldwide are continuously infected with HBV despite the existence of strong vaccination programs.^11^

In Brazil, since 2013, hepatitis B vaccination has been available in the public health system for people up to 49 years of age and age‐insensitive people, including sexologists. Regardless of whether the hepatitis B vaccine is available, vaccination is not universally available, resulting in more people being exposed to infection.^12^ In the Netherlands, a 2002 mass vaccination program reduced the incidence of hepatitis B from 1.8 to 1.2.^13^

### 2.2 Rate of full hepatitis B vaccination

A retrospective cohort study of 42,294 adults in the United Kingdom found that 155,564 (22%) completed three doses of hepatitis B within the recommended 6 months. In the United States, the hepatitis B vaccination coverage was 31.17%. In Brazil, a study of 402 women found that 26.3% had received three doses of hepatitis B vaccine.^14^

## 3 FACTORS INFLUENCING THE FULL HEPATITIS B VACCINATION

### 3.1 Health insurance

Another variable to consider when considering hepatitis B vaccination is health insurance coverage. National surveys have shown that health insurance coverage is fundamentally associated with hepatitis B vaccination.^14^ Therefore, uninsured people have lower health outcomes and survival than insured people.^13^ Similarly, the letter suggests that vaccine providers are reluctant to develop further antibodies and prescribe them to patients unless they are safe for hepatitis B.^15^

Providers typically purchase antibodies in advance and receive refunds to patients after arrangements have been made.^16^ In any case, buying hepatitis B antibodies is not without problems. For example, hepatitis B health insurance for people under the age of 65 is mostly covered by companies (63%), general health programs (13%), or specifically by individuals paying out‐of‐pocket (2%).^17^ Nonetheless, many adults in clandestine custody must meet retention restrictions before they can secure antibodies.^18^ In addition, vaccines secured under open projects reduce hepatitis B vaccination coverage because open suppliers cannot afford the costs of purchasing and organizing antibodies. Open projects (direct procurement) for purchasing vaccines are accessible but restricted.^19^

### 3.2 Place of routine medicinal services

Place of routine medicinal services affects acceptance of hepatitis B vaccine. Some studies suggest that access to health care services frameworks through institutions or other routine welfare service locations is an important determinant in the acceptance of hepatitis B partial vaccination in high‐risk adults. For example, adults who receive deterrent care and HIV testing at centers are more likely to receive hepatitis B vaccination.^20^

Nakwagala and Kagimu evaluated vaccination coverage in high‐risk adults aged 18–49 years.^21^ Another review by Braka et al. found that hepatitis B vaccination was associated with HIV testing, acquisition of hepatitis antibodies, and acceptance of influenza vaccination.^22^ Simonsen et al. report suggests that access to HIV and human services through her STD centers and other health protection agencies could help reduce her HBV transmission in high‐risk gatherings, if innovative projects are implemented.^17^

### 3.3 Use of health care services

Delayed visits to a primary care provider and medical advice may play an important role in hepatitis B vaccination. Pido and Kagimu^24^ reported that a healthcare provider's offer of hepatitis B vaccination was associated with widespread vaccination in men who have sex with men (MSM). Thus, Simonsen et al. showed that her MSM who delayed seeing a doctor likely had hepatitis B vaccination.^17^

Specifically, some studies have reported that MSM who disclose sexual contacts and practices to healthcare providers are more likely to be vaccinated than men who do not disclose sexual contacts and practices.^18^ Conversely, various studies suggest that providing medical information about high‐risk behaviors such as unprotected sex, HIV‐positive status, MSM, or injecting drugs does not actually increase vaccination coverage.

In other words, only 32% of patients treated or being treated for HIV infection were tested for hepatitis B antibodies, even though all were considered at risk for HBV disease. In addition, Simonsen et al. found low vaccination rates (31.6%) among HIV‐infected patients who received antiviral therapy for HIV infection.^17^

### 3.4 Age

It has been shown that age affects the uptake of hepatitis B screening. Grob et al. from their study of 1704 Vietnamese Americans in Northern California and Washington, DC. Underlying reasons for hepatitis B screening were found to be young age, 10+ years of US residency, and lack of familiarity with the Vietnamese language. Subsequently, Grob et al. investigated social and fundamental aspects related to HIV contamination among drug‐administering female sex therapists in the US metropolitan area of Mexico.^20^

### 3.5 Religion

Religious principles affect the acceptance of hepatitis B screening as well as vaccination. Nakwagala and Kagimu studied hepatitis B screening in a Turkish Dutch population in Rotterdam, the Netherlands. This subjective assessment of social determinants found that hepatitis B screening practices may be influenced by societal perceptions of hepatitis B, social standards related to vaccination, social standards related to screening, and social support related to HBV screening. Religious involvement has been observed to be a social issue, particularly identified during her hepatitis B screening.^21^

### 3.6 Education level

Educational levels have been shown to affect hepatitis B infections as well as screening and uptake of vaccination. Braka et al. analyzed symptoms of syphilis, HIV, and hepatitis C in men who had sexual relations with men in Beijing, China. Illness was associated with reduced educational training.^22^ In another study, this training was independent of physical exercise in female sex workers (FSWs). Grob et al. investigated hepatitis B awareness, testing, and information in Vietnamese‐American men and women. School fees in Asians have been observed to be related to HBV testing. Inherently, education has a positive and significant association with HBV screening, and people without adequate secondary education are less likely to be screened.^20^

### 3.7 Marital status

It has been shown that marital status affects the rate of screening for hepatitis B and the uptake of vaccination as married people are more disciplined and focused. Ziraba found that married or cohabiting individuals had more constructive goals and were more likely to participate in screening than unmarried individuals. When they weighed the impact on marriage, they also found that attracting a spouse increased women's overall expectation of screening.^23^

Pido and Kagimu reported on Moldova. In Chisinau, the proportion is double that of Balti. They said they had had a spouse or live‐in partner within the past 12 months. Of these, only 15% in Chisinau and 17% in Balti reported predictable condom use.^24^ In Chisinau, Balti's FSW, three times as many as her, reported having had a casual sex partner in the past 12 months. Of these, 85% in Chisinau (middle of 5) and 55% in Balti (middle of 3) had at least two uncomplicated partners in the previous 12 months. Only 23% in Chisinau and 35% in Balti said they had definitely used a condom with a loose partner in the last 12 months.^24^

### 3.8 Income

Income level affects hepatitis B screening and accessing healthcare services. A benchmark study of an HIV‐positive woman and her HIV‐negative women showed that the two groups of women were socioeconomically equivalent.^20^ Pido and Kagimu investigated hepatitis B awareness, testing, and information in Vietnamese‐American men and women.^24^ It was concluded that Asian family income was associated with HBV testing. Another review by Pido and Kagimu that examined hepatitis B screening among Chinese Americans found that household income was not associated with hepatitis B screening.^24^

However, Pido and Kagim found that poor women were reluctant to approach formal health authorities who use oral and social dissections to assess the health status of HIV‐positive women in Kenya. Economic conditions and transportation costs were also found to contribute to delays and the need to seek medical attention, such as physical examinations.^24^

### 3.9 Social support

Social support is reported to affect the uptake of hepatitis B screening and vaccination. Levin studied hepatitis B screening in a Turkish Dutch population in Rotterdam, the Netherlands. This subjective assessment of social determinants found that social support associated with HBV screening influenced screening, or possibly vaccination behavior.^25^

Social support was also a socio‐social issue identified with hepatitis B screening. One of Shepard's drivers was his social support to get tested for hepatitis B.^26^ Social support has been clearly shown to contribute to getting tested for hepatitis B. Kane investigated barriers in screening for hepatitis B infection in Asian Americans. Social factors such as power, older age, and respect for men in leadership positions have been shown to influence HBV screening. Because of their strong belief in the disease, they may hide their HBV disease from unsupported relatives.^14^

### 3.10 Stigmatization

Stigmatization of sex workers has been reported to affect the hepatitis B screening uptake and vaccination as they hide because of how society will perceive and treat them. A San Francisco review by Canada's Kao noted that sex workers often hide their contributions to sex work and drug use for fear of being judged or treated inappropriately. When they reveal their profession, they are regularly met with disdainful attitudes from staff, dissatisfaction, shame, differences in appearance from other patients, and a sexual and corrupt approach to their work.^27^

According to the World Health Organization,^13^ a meeting of sex workers was set up in Amsterdam to discuss safer sex and distribute condoms to other sex professionals in questionable areas of the city. According to World Health Organization,^28^ the activity has been used to decriminalize, reduce shame and segregation for those who provide or purchase sexual services, and establish basic rights at work, including general health and safety.

### 3.11 Supposition/opinion toward HBV administrations

The awareness of society affects the uptake of hepatitis B screening and vaccination. In a survey on sex workers' vaccination decision‐making and awareness of free hepatitis B vaccination programs, the World Health Organization found that of 259 sex workers encountered at work in the Netherlands, 79% said they were aware of the possibility of hepatitis B vaccination and 63% said they were vaccinated against hepatitis B.^28^ Those who wish to be vaccinated are also screened, but this study did not assess the extent of hepatitis B testing among FSWs.^28^

A World Health Organization survey of 1704 Vietnamese Americans in Northern California and Washington, DC found that only 60% received HBV screening and 26% received HBV vaccination.^28^ The World Health Organization investigated the ebb and flow of screening tests for AIDS, syphilis, and hepatitis B among women in the first trimester of pregnancy living in remote and rugged areas, and investigated their effects. Eighty‐three women were not screened for AIDS, syphilis, or hepatitis B. 42% of pregnant women were not screened because they did not understand the function of screening for AIDS, syphilis, and hepatitis B.^28^

Margolis et al. investigated the awareness and acceptance of hepatitis B screening in Owerri, southeastern Nigeria. They found that while awareness of hepatitis B screening was high at 52.8%, actual levels of screening were low at only 7.1%.^29^ Lack of mindfulness and fear of terrible results were the most common reasons people in this group did not take tests.^29^

## 4 CONCLUSION

While hepatitis B vaccination coverage is low, a significant number of people are not adhering to full hepatitis B vaccination posing a potential hazard to health. So many factors affect hepatitis B screening and vaccination, such as educational level, income level, stigmatization, and many others, which should be addressed for the control of the spread of hepatitis B infection.

## AUTHOR CONTRIBUTIONS

**Emmanuel Ifeanyi Obeagu**: Conceptualization; methodology; resources; supervision; validation; visualization; writing—original draft; writing—review and editing.

## CONFLICT OF INTEREST STATEMENT

The author declares no conflict of interest.

## ETHICS STATEMENT

There are no human participants in this article and informed consent is not applicable.

## TRANSPARENCY STATEMENT

The lead author Emmanuel Ifeanyi Obeagu affirms that this manuscript is an honest, accurate, and transparent account of the study being reported; that no important aspects of the study have been omitted; and that any discrepancies from the study as planned (and, if relevant, registered) have been explained.

## Data Availability

No data were used for the research described in the article.
